# Deregulation of the circadian clock constitutes a significant factor in tumorigenesis: a clockwork cancer. Part II. *In vivo* studies

**DOI:** 10.1080/13102818.2014.925298

**Published:** 2014-07-24

**Authors:** Kristin Uth, Roger Sleigh

**Affiliations:** ^a^ CMCBR, Abertay University, Dundee, Scotland, UK

**Keywords:** circadian clock, regulation, cell cycle, DNA repair, carcinogenesis

## Abstract

The uneventful progression through the cell cycle is closely associated with the rhythm set by the circadian clock machinery, with the S-phase of the cell cycle typically occurring at night. Presence of unrepaired DNA damage may reset the phase of the circadian clock, providing opportunities for damage assessment, repair and/or the induction of pro-apoptotic pathways. The core proteins of the circadian clock regulate directly or indirectly a significant number of genes coding for proteins involved in checkpoint transition, cell proliferation and programmed cell death. Disruption of the circadian rhythm may increase the risk for some multifactorial diseases and conditions, including glucose intolerance, cardiovascular disease and various common cancers. In patients with cancer, chronic circadian misalignment may stimulate the growth of tumours and may modify the outcomes of anticancer therapy. Knowledge about the role of physiological rhythms in human disease may contribute to the field of individualized medicine, specifically, in risk assessment and prognostication of the outcomes in patients with multifactorial disease.

## Abbreviations

NER: Nucleotide excision repair
Per: Period
CNS: Central nervous system
Cry: Cryptochrome

## Introduction

Several drosophila, mouse and rat models with altered or disrupted periodicity regulator genes have already been created.[1–5] The resulting phenotypes may significantly vary with regard to capacity to maintain rhythmicity in absence of entraining cues (from ‘rhythmic albeit phase-shifted’ to ‘completely arrhythmic’) and length of cycle (22–28 hours).[6] Some of the mouse models with disrupted core periodicity genes may exhibit accelerated aging phenotypes of varying severity and cancer proneness.

### *Clock * mouse mutants

Mutations in the *Clock* gene in mice alters the duration of the diurnal cycle in animals housed in constant darkness, although the rhythm is not completely lost.[7] Homozygous *Clock* mutants may exhibit deregulation of feeding rhythmicity and reduced energy expenditure. They are prone to overeating and rapidly develop obesity, hyperglycemia, hypoinsulinemia and hyperlipidemia.[8] *Clock*-deficient mice have shorter-than-normal lifespan and somewhat increased incidence of age-related cataract and dermatitis.[9] The reproductive fitness was moderately decreased both in *Clock*-deficient mice and in carriers of the Δ19 mutation in the *Clock* gene (causing ‘skipping’ of exon 19).[10,11] *Clock*-deficient mice may exhibit increased activity levels, increased sensitization to alcohol and cocaine and increased drug reward compared to wild-type mice,[12] leading to the supposition that the *Clock* protein was involved in the regulation of the dopaminergic system in mammals. The incidence of cancer in *Clock*-deficient mice has not been found to be increased when compared to non-mutant animals.[13]

### *Bmal1* mouse mutants

The loss of the *Bmal1* gene in mice results in immediate loss of circadian rhythmicity when the animals are housed in constant darkness.[6] *Bmal1* mutant mice also exhibit impaired glucose tolerance and decreased insulin secretion that tended to become worse with advancing age.[14] *Bmal1*-deficient mice are sterile and have severely shortened lifespan (27 weeks on the average, compared to 70–150 weeks in normal mice), have lower body weight compared to wild-type mice and also exhibit various traits associated with premature aging such as early development of cataracts, arthropathy, ectopic calcification and loss of muscle and subcutaneous fat.[15–17] Data about cancer-proneness in homozygous *Bmal1* mutant mice is unreliable, as the phenotype of accelerated aging causes early death. Some of the homozygote mice exhibited hyperplasia of the salivary glands and several per cent of them developed lymphoma after gamma-irradiation, although their lifespan was short either way. Heterozygous *Bmal1*-knockout mice exhibit increased cancer-proneness, spontaneous as well as after genotoxic challenge (ionizing radiation).[17]

### *Per and Cry* mouse mutants

In mouse models, genes coding for both *Cry* proteins must be disrupted to produce a phenotype of deregulation of circadian rhythms, as normal functioning of the product of the one gene may partially substitute for the other.[3,18] Only mice with combined *Cry1/Cry2* homozygous knockouts instantly lose the diurnal rhythm when housed in complete darkness, whereas mice lacking the expression of only one of the two proteins exhibit longer or shorter cycle when housed in complete darkness.[3] Mice with targeted disruption of the *Per3* gene exhibit only subtle disturbances of the circadian clock.[19]

The circadian clock is adjustable by food cues. Mice deficient in *Per2* are unable to predict and anticipate the approximate time when food is normally available.[19,20] Mice with mutant *Npas2* or deficient for *Cry1* and *Cry2* are impaired in food-associated entrainment of the circadian clock.[20]

*Cry1* homozygous knockout mice, double *Cry1/Cry2* knockout mice and *Per2* mutant homozygous mutant mice exhibit increased rates of spontaneous tumours and tumours developing after genotoxic challenge (ionizing radiation).[17,21,22,23] The circadian pattern of expression of some genes coding for proteins involved in the control of the progression in the cell cycle (c-Myc, Cyclin D1, Cyclin A, Mdm-2) is grossly deregulated in mice with mutant *Per2*.[22,23]

*Cry1/Cry2* deficient double mutant mice that also carry inactivated *Tp53* gene copies exhibit longer cancer-free survival and somewhat extended lifespan than p53-deficient mice without *Cry* mutations, although both groups virtually never reach the lifespan of normal mice.[24] This is believed to be associated with increased propensity toward apoptosis by the p53-independent mechanism, conferred by deficiency of *Cry* proteins.[24]

## Human phenotypes associated with carriership of mutations or polymorphisms in clock machinery genes

Accelerated aging is a prominent feature in the animal models of deficiency or modification of core clock genes. It is therefore possible that the oscillating circadian clock may be an adjusting factor for the general molecular clock of aging. Aging is currently viewed as a preprogrammed mechanism, rather than a simple product of wear and tear of tissues and organs.[25] The rate of attrition of telomere ends is currently viewed as a timing mechanism of the unidirectional (hourglass) type.[26] For single cells, telomere attrition rate is a marker for the cell's proximity to replicative senescence. On a higher level, however, telomere length and rate of attrition of telomere ends provide a basis for the assessment of the rate of aging of tissues, organs and organisms. Shortening of telomere ends as a designated mechanism for induction of aging has been intensively studied, as it provides quantitative data that may be measured and compared between experiments.[27,28] Research has already shown that the circadian pattern of expression of clock genes becomes markedly impaired in senescent cells.[29,30] This, however, was shown to be remedied (albeit temporarily) by telomere lengthening. It has been proposed that the functioning of the oscillator clocks is dependent on the ‘time’ shown by the unidirectional clock of aging, with the peripheral clocks in aged tissues becoming less responsive to the signals sent by the master clock.[29]

In humans, the presence of variant alleles of genes coding for proteins functioning in the circadian clock may cause sleep phase shift syndromes. These are usually quite benign and only very rarely interfere significantly with the normal life of the affected individual. The Ser662Gly mutation (rs121908635) in the *PER2* gene is known to cause advanced sleep phase syndrome 1, manifested by the need to go to bed very early in the evening (6–8 pm) and spontaneous awakening very early in the morning (3–4 am).[31] The ‘natural short sleeper’ human phenotype is associated with heterozygous carriership of the mutation Pro385Arg in the *DEC2* gene.[32,33] Carrier individuals require fewer hours of sleep per 24 h (on the average, 6 hours) to be completely rested than what is considered normal in the general population (7–9 h per 24 h). Presence of the *DEC2* Pro385Arg mutation does not seem to be associated with any adverse effects.

One noncoding polymorphism in the 3′-UTR of the human *CLOCK* gene (a T-to-C transition, rs1801260) is associated with adult attention-deficit and hyperactivity disorder (ADHD).[34]

Several inherited polymorphisms in core clock genes may be associated with increased risk for development of various tumours. The 311T>C single-nucleotide polymorphism in the human *CLOCK1* gene have been associated with significantly increased susceptibility to colorectal carcinoma.[35] The 5-repeat variant allele of the 4/5 repeat polymorphism in the human *PER3* gene is associated with almost twofold increased risk for breast cancer in premenopausal women.[36] The Ala394Thr polymorphism (rs2305160) in the coding sequence (specifically, in the PAS domain) of the human *NPAS2* gene has also been found to increase the risk for non-Hodgkin's lymphoma.[37]

Current research data show that the disruption of the circadian cycle may increase the risk for common diseases and conditions (diabetes, cancer, cardiovascular disease) and/or accelerate their progression. Of course, as in all diseases with multifactorial genesis, the presence of an additional risk factor does not mean that the individual will definitely develop the disease or condition, only that in its presence the risk is increased compared to the general population.[38] Considering that the factors disrupting the circadian rhythm are very common, especially in industrialized countries (light at night, insufficient lighting during the day, evening and night shift work and/or rotating shifts, major meal for the day consumed in the evening hours, frequent transmeridional flights, etc.), it could be expected that the health impact would continue to increase in the future. Lifestyle and jobs including disruption of the day-night rhythm (specifically, night shift work) may be associated with increased incidence of colorectal carcinoma, breast, lung and prostate cancer in man.[39–43] The majority of the studies on the impact of disruption of circadian rhythms on human health were conducted in female controls (predominantly nurses), therefore, one cannot exclude gender-specific differences (at least for some cancers). There is also the fact that nurses, as health workers, were motivated for continuing participation in these studies. The attitude of the participating individuals towards any medical study (and, especially, studies related to cancer medicine) may seriously affect the study, in terms of attrition rate, reliability of the personal data provided by the study objects, and the potential health benefits (opportunities to care for one's health in a more efficient manner – e.g. having more regular checkups).[44] Medical personnel are, on the whole, more likely to stick to the study from the beginning to the end, fill the questionnaires accurately (because of pre-existing knowledge about the problem under study) and have their medical checkups regularly, allowing for early diagnosis and more precise risk assessment. In a 2007 press release the International Agency for research on Cancer (IARC) pronounced that ‘…Shiftwork that involves circadian disruption is probably carcinogenic to humans…’ [http://www.iarc.fr/en/media-centre/pr/2007/pr180.html]. The risk has been found to be especially high for those on rotating night shifts, not allowing for adaption of the circadian clock to the timing of the subjective day and the subjective night. The increased risk for lung cancer in night shift workers was, however, modifiable by environmental factors (specifically, smoking)[42], and was not reported in all studied populations.[45]

The association between disruption of the diurnal rhythm and breast cancer has been particularly well studied. It has been speculated that disrupted circadian rhythms (lack of natural (sun-spectrum) lighting or simply insufficient lighting during the day and/or artificial lighting at night) may be at least partially responsible for the increasing rates of breast cancer in industrialized countries. There have been reports about lower overall incidence of cancer in people with total visual blindness (could not perceive light at all) compared to the general population.[46] The association was specifically strong for mammary gland cancer (over twofold risk reduction in individuals with total visual blindness than in the general population),[47,48] but the risk for prostate cancer was also found to be lower among totally blind men.[49] Since hormone release usually follows a circadian rhythm, and the majority of breast and prostate cancers are hormone-dependent, these results are not unexpected.

Disruption of circadian rhythms may modify the course of neoplastic disease. In recent studies, light at night promoted tumour growth in mouse models with breast cancer.[50,51] In human patients with breast carcinoma, daily bedtime misalignment (i.e. misalignment between preferred bedtime and actual bedtime) was shown to be associated with more rapid cancer progression.[52] It has even proposed that the risk for melanoma may be increased in individuals habitually exposed to excess light in the evening and night.[53]

Mutations in circadian clock genes have been observed in tumour tissue – specifically, in colorectal cancer, breast cancer, prostate cancer and thyroid carcinoma.[54–56] The expression of *DEC1* is down-regulated or absent in >50% of oesophageal cancers in man.[57] Disruption of the normal circadian cycle may be implemented on epigenetic level in tumour cells. One study of tumour tissues from breast cancer patients found hypermethylation on the promoters of *PER1*, *PER2*, *CRY1* or *BMAL1* genes in about 70% of the cases, compared to <50% methylation in non-cancerous tissues.[54] In the same study, homogeneous (non-rhythmic) expression of *PER2* or *BMAL1* was significantly associated with lymph node metastasis and poorer prognosis for the patient. *PER1* and *PER2* genes are currently considered to be true tumour suppressor genes, as decreased expression of either (or both) has been reported in several types of human cancers (pancreatic cancer, renal cancer, head and neck cancers)[58–60] and reduced *PER1* and *PER2* expression was recently shown to be an independent predictor of poorer prognosis in patients with gastric cancer.[61] In 2009, it was proposed that the growth rate of breast cancer corresponded to the rhythm set by the circadian clock.[62] Down-regulation of *Per2* resulted in increases in the levels of Cyclin D and Cyclin E and accelerated tumour growth *in vivo* [63] whereas induced overexpression of either *Per1* or *Per2* has been shown to inhibit the growth of cancer cells and increase their apoptotic rate.[64] In patients with chronic lymphocytic leukaemia (CLL), the expression of *BMAL1*, *PER1* and *PER2* was found to be significantly down-regulated in comparison to healthy controls whereas the expression of the proto-oncogene *c-MYC* and Cyclin D1 was significantly up-regulated.[65]

The expression profile of the core clock proteins may affect the outcomes in patients with cancer. Among patients with CLL, different *CRY1* expression levels and *CRY1/PER2* expression ratio were associated with different clinical course (and, respectively, very different outcomes), with epigenetically silenced *CRY1* gene usually associated with indolent disease, needing treatment only in its very late phases, if ever.[66,67] Thus, the levels of expression of circadian clock genes may be used as auxiliary markers in the prognostic panel in CLL.[67–69] The overall survival of patients with colorectal cancer with high *BMAL1* levels in the primary tumour was significantly longer (1.5 times) than that of patients with tumours with low *BMAL1* levels; and the progression-free survival was more than two times higher in patients with high *BMAL1* expression than that in patients with low expression.[70] High levels of expression of the negative regulator of the core feedback loop *CRY1* were associated with poorer overall survival in patients with colorectal cancer.[71]

The levels of expression of the genes of the circadian core clock and the accessory proteins may predict sensitivity to anticancer therapies and/or provide information about the opportunities for therapeutic intervention. *BMAL1* overexpression was shown to inhibit the growth of human colorectal cancer cell lines, increasing their sensitivity to genotoxic agents (oxaliplatin).[70] It has been shown that Tim-depleted cancer cells may become sensitive to doxorubicin (a topoisomerase II inhibitor), making Tim1 a potential anticancer target in therapies based on ATM/ATR damage response pathway inhibition.[72–74]

Currently, there is a massive research effort concentrated at studying the effects of timing of anticancer therapies around the circadian rhythm on the chances of achieving the maximal possible therapeutic effect (cancer chronotherapy).[75] Most currently used anticancer agents work by infliction of DNA damage (genotoxicity). Their effect is strongest in rapidly dividing cells, as tumour cells are, but may affect the functioning of non-tumour cells with naturally rapid turnover as well. In principle, anticancer therapies are considered appropriate if they fulfil two requirements: (1) they produce a good objective response (tumour regression, slowing down the growth of the tumour) and (2) their use is coupled with minimal adverse effects for the patient. It is now agreed that anticancer therapy needs a considerable amount of customization to achieve the optimal balance of these two factors for the particular patient.[76] Basically, in cancer chronotherapy, the application of the genotoxic agent/s is timed to the 24-h circadian variations (and, sometimes, to intradian (8–12 h) variations) in the mitotic index of cancer cells. Timing of genotoxic therapy to the specific time of the day when it is supposed to produce the greatest amount of damage to specific type of tumour cells may be expected to be associated with greater treatment response. At the same time, as the mitotic index of cancer and normal cells of the same tissue may peak at different times of the day, it could be expected that the associated toxicities for the normal tissues would be lower (least toxic times of chemotherapy). For example, a study among patients with ovarian cancer showed that the circadian peak in DNA synthesis for tumour cells was found between noon and 4 pm, which was ≈12 hours off the peak of DNA synthesis in non-tumour cells (usually occurring at night).[77] Similarly, in patients with non-Hodgkin lymphoma, the peak of DNA synthesis in affected lymph nodes occurred at night, whereas the highest levels of DNA synthesis in healthy cells in the bone marrow were observed in the early hours of the afternoon.[78] So far, the trials of cancer chronotherapy have shown that timing of genotoxic therapies within the circadian cycle produces significantly lower rates of severe adverse effects than ‘conventionally timed’ therapy.[79–81] Indeed, undergoing cancer chronotherapy may mean significantly more treatment-related time spent in hospital, as the optimal time for drug administration may be late at night or very early in the morning, but since the outcomes may be objectively better than in conventionally timed therapy, there is every chance that chronotherapy for cancer may find broader applications in the near future. Obtaining data about lifestyle traits and habits possibly disrupting the circadian rhythm as well as testing for the presence of variants of the core clock genes may make a valuable addition to the panels of assessment of individual repair capacity, alongside polymorphisms in genes coding for proteins of DNA repair and maintenance of genome integrity and unscheduled DNA synthesis, especially in patients with cancer.[28,76,82–84]

The circadian clock may play a role in the pathogenesis of multifactorial diseases and conditions other than cancer. For example, deregulated expression of *Bmal1* and *Cry1* was detected in animal models of traumatic brain injury.[85] The sleep-wake cycle is grossly disturbed in patients with dementia of Alzheimer's type.[86,87] It has been hypothesized that the regulation of the peripheral circadian clocks (e.g. in the cardiovascular system) may become more and more difficult with age.[29] This impaired peripheral circadian rhythm may produce deregulated expression of tissue-specific clock-controlled genes, resulting in cardiovascular disease (e.g. plasminogen activator inhibitor-1, vascular endothelial growth factor and others).[29,88,89]

It is well known that the occurrence of stroke usually peaks in the morning (roughly between 6 and 12 am).[90] The same is valid for myocardial infarction and sudden cardiac death, where the risk for incidents is highest in the several hours after morning awakening.[91] Neuronal susceptibility to ischemic events seems to follow a circadian pattern.[92] In *Per1-*deficient-mice with cerebral ischaemia, higher rates of neuronal injury have been observed than in non-mutant ischaemic controls.[93] CNS cells exhibit variations in the activation of apoptosis markers (expression of caspases-3, -8 and -9) in response to cerebral ischemia induced at different times of day.[93,94] Ischemia is typically associated with increased levels of oxidative stress. It is possible that the disruption of the circadian rhythm associated with oxidative DNA damage may impair the functioning of the system for the assessment of the scale and scope of the damage, with the result that damaged cells in clock-disrupted models are more likely to be routed to the apoptotic pathway than in controls (where the same amount of DNA damage might have been assessed as ‘repairable’). Carriership of variant alleles associated with increased levels of unrepaired DNA damage and the associated propensity toward apoptosis in the vascular wall may contribute to the ‘morning peak’, and to the disruption of the circadian cycle commonly seen in stroke patients in the period immediately following the incident.[95,96]

Circadian misalignment has been shown to increase insulin resistance and augment expression of inflammatory markers in diabetes type 2.[97] The decline in beta-cell function seen in the progression of diabetes type 2 may be accelerated by the disruption of circadian rhythms and accelerate development of diabetes type 2.[98,99]

## Conclusions

Circadian clock dysfunction plays a role in the pathogenesis of many common multifactorial diseases and conditions, including glucose intolerance, cardiovascular disease and cancer. The core clock proteins may directly or indirectly modulate the expression of proteins functioning in the progression of the cell cycle. Disruption of the rhythm set by the internal clock may increase the risk for development of disease, aggravate the course of pre-existing conditions and modulate the outcomes of anticancer therapy. Knowledge about the mechanisms governing the maintenance of circadian rhythms and recovery of this rhythm after disruption may provide opportunities for informed lifestyle modification, more efficient therapeutic intervention and may assist in more individualized selection of anticancer therapies and schedules as well the prognostication of outcomes in patients with multifactorial diseases. 
